# Association between physical activity intensity and self-reported emphysema: A cross-sectional study using NHANES

**DOI:** 10.1371/journal.pone.0362852

**Published:** 2026-11-13

**Authors:** Qiao Wei, Haiyan Song, Li Jiang, Xiuqin Li, Jihua Zhu

**Affiliations:** 1 Bozhou Hospital affiliated to Anhui Medical University, Bozhou Peoples Hospital, Bozhou, Anhui, China; 2 Zhejiang University School of Medicine, Affiliated Childrens Hospital of Zhejiang University, Hangzhou, Zhejiang, China; Xuzhou Central Hospital, The Xuzhou School of Clinical Medicine of Nanjing Medical University, CHINA

## Abstract

**Background:**

Emphysema is a significant public health issue. Physical activity (PA) has been suggested to be associated with respiratory health; however, the relationship between PA intensity and emphysema remains insufficiently characterized.

**Methods:**

This cross-sectional study utilized data from the National Health and Nutrition Examination Survey (NHANES) from 2007 to 2016. Emphysema was defined based on self-reported physician diagnosis. Multivariable logistic regression models were used to examine the association between different levels of PA and the odds of self-reported emphysema among older adults, adjusting for demographic characteristics, lifestyle factors, and comorbidities.

**Results:**

Higher levels of physical activity, particularly ≥3000 MET-minutes per week, were associated with lower odds of self-reported emphysema. A dose–response relationship was observed, with increasing PA levels associated with progressively lower odds of self-reported emphysema.

**Conclusions:**

Higher physical activity levels were associated with lower odds of self-reported emphysema in this cross-sectional analysis. These findings highlight a potential association between PA and self-reported emphysema; however, causal inference cannot be established. Prospective studies are warranted to further evaluate this relationship and explore underlying mechanisms.

## Introduction

Emphysema, one of the primary manifestations of chronic obstructive pulmonary disease (COPD), is characterized by the abnormal, permanent enlargement of the airspaces, leading to impaired gas exchange, airflow limitation, and altered airway dynamics [1]. According to the World Health Organization, COPD is among the leading causes of mortality worldwide, particularly among older adults. The burden of emphysema is especially high in long-term smokers and the elderly population [2]. In addition, previous studies have reported that emphysema is associated with an increased likelihood of lung cancer, further contributing to disease burden [1,3,4]. However, current management strategies for emphysema primarily rely on pharmacological and surgical interventions, which may have limited effectiveness and are often associated with substantial risk, particularly in advanced stages [5]. Therefore, identifying modifiable lifestyle factors remains an important area of research.

In recent years, physical exercise, as a form of non-pharmacological intervention, has increasingly been recognized for its role in the management of chronic diseases. Physical activity has been associated with alleviation of symptoms and improved quality of life for patients with emphysema through mechanisms such as enhancing cardiopulmonary function, strengthening respiratory muscle strength, and reducing airway inflammation [6]. Additionally, physical activity has also been associated with better psychological health, including reduced symptoms of depression and anxiety, which are common among patients with emphysema [7]. The metabolic equivalent of task (MET), as a standard unit for assessing the intensity of physical activities, has been used to quantify physical activity levels in epidemiological studies [8,9]. Research indicates that maintaining a moderate or higher level of MET is associated with improved cardiopulmonary function and muscle mass, and may be linked to lower prevalence of chronic diseases, including emphysema [10,11].

Based on data from the National Health and Nutrition Examination Survey (NHANES), this study systematically analyzes the association between physical activity intensity and self-reported emphysema among the elderly. Employing rigorous statistical models, we adjusted for a variety of potential confounding factors, including demographic characteristics, metabolic status, lifestyle, and comorbid conditions, to further examine the relationship between physical activity levels and self-reported emphysema. This research aims to fill a gap in this field, providing population-based evidence on the association between physical activity and self-reported emphysema, and may contribute to informing public health policies and personalized health intervention strategies.

## Materials and methods

### Study design

This was a cross-sectional study based on NHANES data. This study employed data derived from the National Health and Nutrition Examination Survey (NHANES), a nationally representative, stratified, multistage probability survey administered by the Centers for Disease Control and Prevention (CDC). The NHANES program is designed to rigorously evaluate the health and nutritional status of the noninstitutionalized U.S. population, employing comprehensive methods including structured interviews, detailed physical examinations, and laboratory assessments conducted at Mobile Examination Centers (MEC). Participation in NHANES is contingent upon the provision of informed consent, with ethical oversight provided by the National Center for Health Statistics (NCHS) Ethics Review Committee. This study used publicly available de-identified NHANES data and was therefore exempt from additional ethical approval. The NHANES repository is openly accessible and can be consulted at https://www.cdc.gov/nchs/nhanes/index.htm.

### Study population

For this analysis, we utilized data from continuous NHANES survey cycles conducted biennially between 2007 and 2016. These datasets provided detailed information regarding physical activity (PA) and the prevalence of self-reported emphysema. The initial cross-sectional cohort included 50,588 individuals. Participants under the age of 60 years (N = 40,828), those with missing data for both independent and dependent variables (N = 24), and individuals with incomplete covariate information (N = 2,858) were excluded, resulting in a final analytical cohort comprising 6,878 participants (Fig 1).

**Fig 1:**
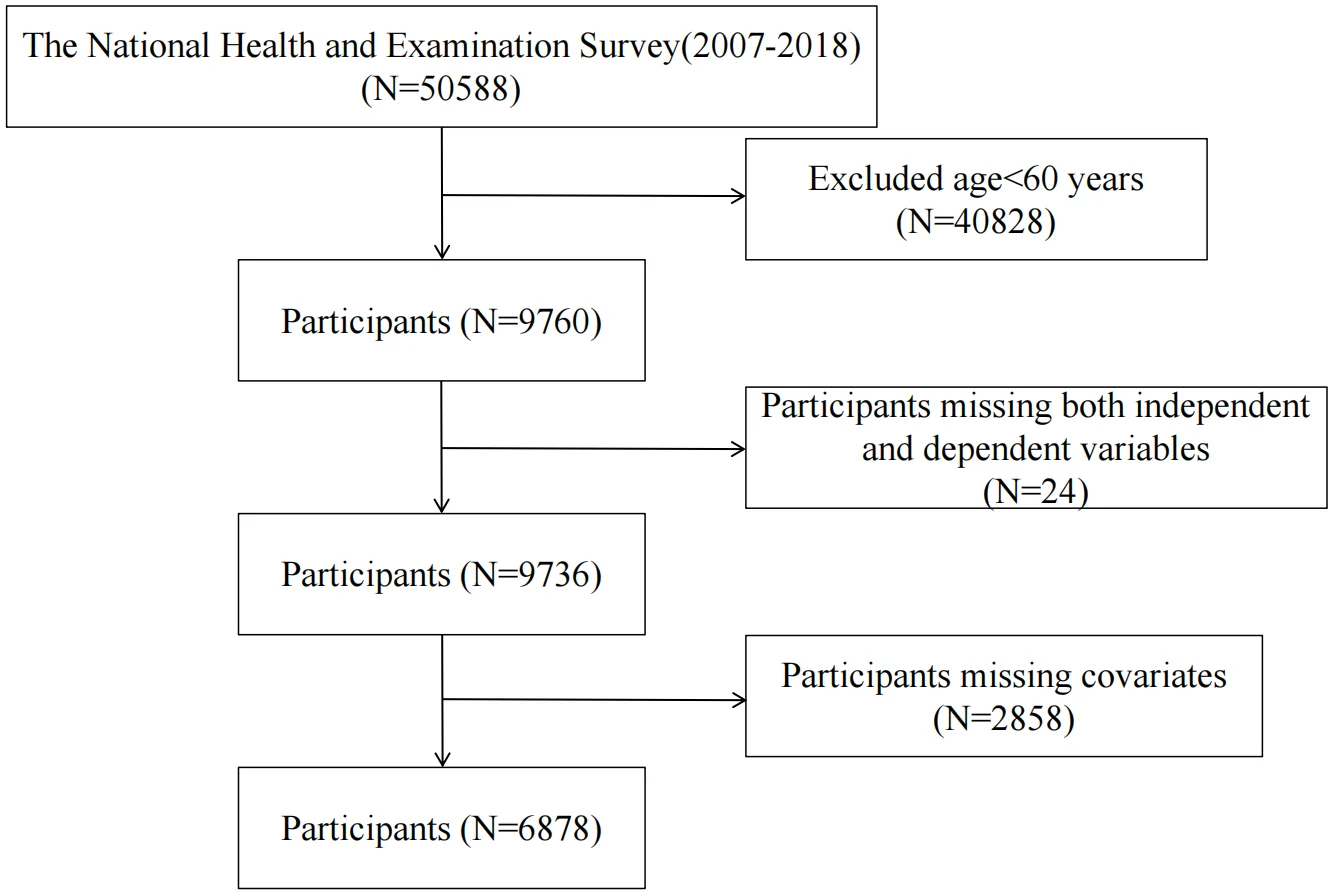
Flowchart of participant selection for the study. Participants were selected from the National Health and Nutrition Examination Survey (NHANES). Of the 50,588 participants initially assessed, 40,828 were excluded because they were younger than 60 years, 24 because of missing data on both physical activity and emphysema status, and 2,858 because of incomplete covariate data. The final analytical sample included 6,878 adults aged 60 years or older.

### Assessment of independent variable

The assessment of PA employed a questionnaire adapted from the Global Physical Activity Questionnaire (GPAQ), collecting participant-level data via structured interviews. PA was classified into five distinct domains: vigorous occupational activity, moderate occupational activity, active transport such as walking or cycling, vigorous leisure-time activity, and moderate leisure-time activity. Vigorous-intensity activities and moderate-intensity/transport activities were assigned metabolic equivalent of task (MET) values of 8.0 and 4.0, respectively. The MET for each activity type was determined by multiplying its reported duration by the corresponding activity score, and the total weekly MET was calculated by summing the METs for all reported activities.

Following the guidelines issued by the CDC and the American College of Sports Medicine (ACSM), which recommend a minimum threshold of 600 MET minutes per week for an adequate evaluation of PA, participants’ weekly METs were stratified into four categories (<600, ≥ 600 to <1800, ≥ 1800 to <3000, and ≥3000) [12]. This stratification facilitated an analysis of the association and dose-response relationship between PA levels and self-reported emphysema.

### Assessment of covariates

A comprehensive set of covariates, informed by prior literature, was assessed for their potential association with the study outcomes. These covariates included age, sex, race or ethnicity, educational attainment, marital status, family income-to-poverty ratio (PIR), body mass index (BMI), levels of total cholesterol and triglycerides, serum creatinine, diabetes mellitus (DM), hypertension, smoking behavior, and alcohol consumption. Family income was categorized into low (PIR ≤ 1.3), medium (PIR > 1.3 to 3.5), and high (PIR > 3.5), consistent with U.S. federal standards [13]. BMI was calculated as weight in kilograms divided by the square of height in meters and classified participants as normal weight (18.5 to <25 kg/m²), overweight (25 to <30 kg/m²), or obese (≥30 kg/m²). Smoking status was defined as never smokers (individuals who had smoked fewer than 100 cigarettes in their lifetime) and ever smokers (those who had smoked at least 100 cigarettes). Alcohol consumption was categorized as never drinkers (fewer than 12 alcoholic drinks per year) and drinkers (12 or more alcoholic drinks annually). Hypertension was identified by a clinical diagnosis, use of antihypertensive medication, or three consecutive blood pressure readings showing systolic blood pressure ≥140 mmHg or diastolic blood pressure ≥90 mmHg [14]. DM was defined by a diagnosis of hyperglycemia, ongoing antidiabetic treatment, or laboratory findings consistent with the condition, including hemoglobin A1c ≥ 6.5%, fasting glucose ≥126 mg/dL, or a 2-hour postprandial glucose level ≥200 mg/dL [15].

### Assessment of outcome variable

Emphysema status was determined based on self-reported physician diagnosis, defined by participants’ affirmative response to the question, “Has a doctor or other health professional ever told you that you had emphysema?”, as recorded in the NHANES medical conditions questionnaire.

### Statistical analysis

Categorical variables were presented as proportions (%), while continuous variables were reported as mean (±SD) or median (IQR), depending on the distribution characteristics. The Kruskal-Wallis test was used to analyze skewed data, whereas one-way ANOVA was employed for normally distributed variables. Categorical variables were compared using the chi-square test. The association between PA and self-reported emphysema was assessed using multivariate logistic regression models. Adjustments for covariates were introduced progressively in three models: Model 1 was unadjusted; Model 2 adjusted for age, gender, and race; and Model 3 included further adjustments for education level, marital status, PIR, BMI, total cholesterol, triglycerides, creatinine, DM, hypertension, smoking status, and alcohol use. To explore potential nonlinear associations between total PA MET-min/week (treated as a continuous variable) and the likelihood of self-reported emphysema, a restricted cubic spline (RCS) analysis with three knots was performed. Subgroup analyses examined the relationship between PA and self-reported emphysema across stratified factors, including sex (male or female), alcohol consumption (yes or no), smoking status (never, former, or current), and BMI categories (normal weight, overweight, or obese). These variables were also analyzed for their role as potential effect modifiers. Statistical significance was defined by a two-sided P-value <0.05. All analyses were conducted using R software, version 4.3.2 (http://www.R-project.org; The R Foundation).

### External validation using CHARLS dataset

To evaluate the external validity of our findings, we conducted a supplementary analysis using data from the China Health and Retirement Longitudinal Study (CHARLS 2015 wave). CHARLS is a nationally representative cohort of middle-aged and older adults in China. We applied the same physical activity categorization (in MET-min/week: < 600, 600– < 1800, 1800– < 3000, and ≥3000) and covariate adjustment strategy as in the NHANES analysis. Logistic regression models were used to estimate the association between total physical activity and self-reported lung disease (emphysema or chronic lung condition) in CHARLS. This external validation was designed to assess the robustness and generalizability of the observed associations across populations with different ethnic and sociodemographic backgrounds.

## Results

### Baseline characteristics of study participants

The baseline characteristics of the 6,878 participants, categorized by total PA levels in MET-min/week, are detailed in Table 1. The median age of the cohort was 69 years (IQR, 64–76), with older individuals more frequently represented in the lower PA categories (p < 0.001). A higher proportion of males (49.89%), individuals with greater income (PIR > 3.5, 35.23%), and those with advanced education (college degree or higher, 25.62%) were observed in the most active group (p < 0.001 for all comparisons). Participants with obesity (31.08%), hypertension, and diabetes were disproportionately represented in the least active groups (p < 0.001). Significant differences were also noted in smoking and alcohol consumption patterns, with fewer current smokers and a higher prevalence of alcohol consumers among those in the higher PA categories (p = 0.023 and p < 0.001, respectively). The overall prevalence of self-reported emphysema was 4.45%, and lower prevalence was observed with increasing PA levels (p < 0.001). These findings suggest that higher PA levels are associated with more favorable sociodemographic and clinical profiles.

**Table 1:** Baseline characteristics of participants.

Characteristic	Total physical activity MET-min/week					
	Total (n = 6878)	<600(n = 3664)	≥600and <1800 (n = 1394)	≥1800and <3000 (n = 571)	≥3000 (n = 1249)	p Value
Age,year,(IQR)	69 [64, 76]	71 [64, 78]	69 [63, 76]	68[63, 74.5]	67[63, 73]	<0.001
Gender,n(%)						<0.001
Male	3431 (49.88)	1598 (43.61)	707 (50.72)	326 (57.09)	800 (64.05)	
Female	3447 (50.12)	2066 (56.39)	687 (49.28)	245 (42.91)	449 (35.95)	
Race,n(%)						<0.001
Mexican American	832 (12.10)	487 (13.29)	140 (10.04)	56 (9.81)	149 (11.93)	
Other Hispanic	687 (9.99)	372 (10.15)	131 (9.40)	46 (8.06)	138 (11.05)	
Non-Hispanic White	3573 (51.95)	1835 (50.08)	756 (54.23)	317 (55.52)	665 (53.24)	
Non-Hispanic Black	1312 (19.08)	748 (20.41)	248 (17.79)	98 (17.16)	218 (17.45)	
Other Race	474 (6.89)	222 (6.06)	119 (8.54)	54 (9.46)	79 (6.33)	
PIR,n(%)						<0.001
<1.3	2046 (29.75)	1274 (34.77)	330 (23.67)	133 (23.29)	309 (24.74)	
1.3-3.5	2826 (41.09)	1532 (41.81)	575 (41.25)	219 (38.35)	500 (40.03)	
>3.5	2006 (29.17)	858 (23.42)	489 (35.08)	219 (38.35)	440 (35.23)	
Educational levels,n(%)						<0.001
Less Than 9th Grade	907 (13.19)	578 (15.78)	134 (9.61)	58 (10.16)	137 (10.97)	
9-11th Grade	949 (13.80)	585 (15.97)	157 (11.26)	58 (10.16)	149 (11.93)	
High School Grad	1671 (24.29)	942 (25.71)	313 (22.45)	124 (21.72)	292 (23.38)	
Some College or AA degree	1804 (26.23)	909 (24.81)	395 (28.34)	149 (26.09)	351 (28.10)	
College Graduate or above	1547 (22.49)	650 (17.74)	395 (28.34)	182 (31.87)	320 (25.62)	
BMI,n(%)						<0.001
<25	1739 (25.28)	834 (22.76)	415 (29.77)	149 (26.09)	341 (27.30)	
≥25 and <30	2532 (36.81)	1299 (35.45)	504 (36.15)	234 (40.98)	495 (39.63)	
≥30	2607 (37.90)	1531 (41.78)	475 (34.07)	188 (32.92)	413 (33.07)	
Marital status,n(%)						<0.001
Married or living with a partner	4101 (59.62)	2060 (56.22)	850 (60.98)	352 (61.65)	839 (67.17)	
Widowed or Separated	2338 (33.99)	1369 (37.36)	448 (32.14)	176 (30.82)	345 (27.62)	
Divorced or Never married	439 (6.38)	235 (6.41)	96 (6.89)	43 (7.53)	65 (5.20)	
Alcohol consumption,n(%)						<0.001
Yes	4513 (65.62)	2260 (61.68)	939 (67.36)	411 (71.98)	903 (72.30)	
No	2365 (34.38)	1404 (38.32)	455 (32.64)	160 (28.02)	346 (27.70)	
Smoking status,n(%)						0.023
Yes	3543 (51.51)	1888 (51.53)	680 (48.78)	291 (50.96)	684 (54.76)	
No	3335 (48.49)	1776 (48.47)	714 (51.22)	280 (49.04)	565 (45.24)	
Total cholesterol (mg/dl)(mean SD)	192.45 (44.11)	191.41 (44.66)	193.74 (45.21)	192.05 (41.77)	194.24 (42.19)	0.148
Triglycerides (mg/dl)(mean SD)	156.82 (104.33)	160.04 (101.82)	152.63 (102.89)	152.86 (97.76)	153.87 (115.31)	0.056
Creatinine(mg/dL)(mean SD)	1.01 (0.58)	1.04 (0.66)	0.99 (0.56)	0.99 (0.48)	0.97 (0.28)	<0.001
Hypertension,n(%)						<0.001
No	2423 (35.23)	1142 (31.17)	519 (37.23)	219 (38.35)	543 (43.47)	
Yes	4455 (64.77)	2522 (68.83)	875 (62.77)	352 (61.65)	706 (56.53)	
Diabetes,n(%)						<0.001
No	5740 (83.45)	2978 (81.28)	1169 (83.86)	486 (85.11)	1107 (88.63)	
Yes	1138 (16.55)	686 (18.72)	225 (16.14)	85 (14.89)	142 (11.37)	
Self-reported emphysema ,n(%)						<0.001
No	6572 (95.55)	3459 (94.41)	1345 (96.48)	553 (96.85)	1215 (97.28)	
Yes	306 (4.45)	205 (5.59)	49 (3.52)	18 (3.15)	34 (2.72)	

### Association between PA and self-reported emphysema

The association between PA levels and self-reported emphysema, as analyzed through multivariable logistic regression models, is summarized in Table 2. In the unadjusted analysis (Model 1), higher levels of PA were significantly associated with lower odds of self-reported emphysema. Participants engaging in ≥600 MET-min/week had an odds ratio (OR) of 0.56 (95% CI, 0.36–0.85; p = 0.008), with the strongest inverse association observed in those exceeding 3000 MET-min/week (OR, 0.40; 95% CI, 0.24–0.65; p < 0.001). This inverse relationship demonstrated a significant dose-response trend (p for trend < 0.001).

**Table 2:** Multivariable logistic regression analyses of physical activity and self-reported emphysema.

Total physical activity MET-min/week	Model 1		Model 2		Model 3	
	OR(95%CI)	P-Value	OR(95%CI)	P-Value	OR(95%CI)	P-Value
<600	Reference		Reference		Reference	
≥600and <1800	0.56(0.36,0.85)	P = 0.008	0.52(0.34,0.79)	P = 0.003	0.69(0.44,1.08)	P = 0.099
≥1800and <3000	0.56(0.30,1.05)	P = 0.070	0.49(0.25,0.94)	P = 0.033	0.61(0.31,1.22)	P = 0.156
≥3000	0.40(0.24,0.65)	P < 0.001	0.34(0.20,0.58)	P < 0.001	0.41(0.23,0.72)	P = 0.002
P for trend	P < 0.001		P < 0.001		P = 0.003	

Model 1: non-adjusted.

Model 2: adjusted for age, gender, race.

Model 3: adjusted for age, gender, race, education levels, marital status, PIR, BMI, smoking status, alcohol consumption, total cholesterol, triglycerides, creatinine, hypertension, diabetes.

After adjusting for age, sex, and race in Model 2, the inverse association between PA and self-reported emphysema remained evident. Participants achieving ≥600 MET-min/week exhibited an OR of 0.52 (95% CI, 0.34–0.79; p = 0.003), while those engaging in >3000 MET-min/week showed lower odds of self-reported emphysema, with an OR of 0.34 (95% CI, 0.20–0.58; p < 0.001). The dose-response relationship across PA levels continued to be significant (p for trend < 0.001).

In the fully adjusted model (Model 3), which accounted for additional covariates including education, marital status, PIR, BMI, smoking status, alcohol consumption, total cholesterol, triglycerides, creatinine, hypertension, and diabetes, the association remained significant only for the highest PA category (>3000 MET-min/week). This group showed an OR of 0.41 (95% CI, 0.23–0.72; p = 0.002), with the trend across PA levels maintaining statistical significance (p for trend = 0.003). These findings indicate a dose-dependent inverse association between PA and self-reported emphysema, particularly at higher levels of activity, even after comprehensive adjustment for potential confounders.

### RCS analysis

The RCS analysis, modeling PA as a continuous variable, demonstrated a nonlinear association between total PA MET-min/week and the odds of self-reported emphysema (Fig 2). The overall relationship was statistically significant (p < 0.001), with further evidence supporting a nonlinear pattern (p for nonlinearity = 0.008).

**Fig 2:**
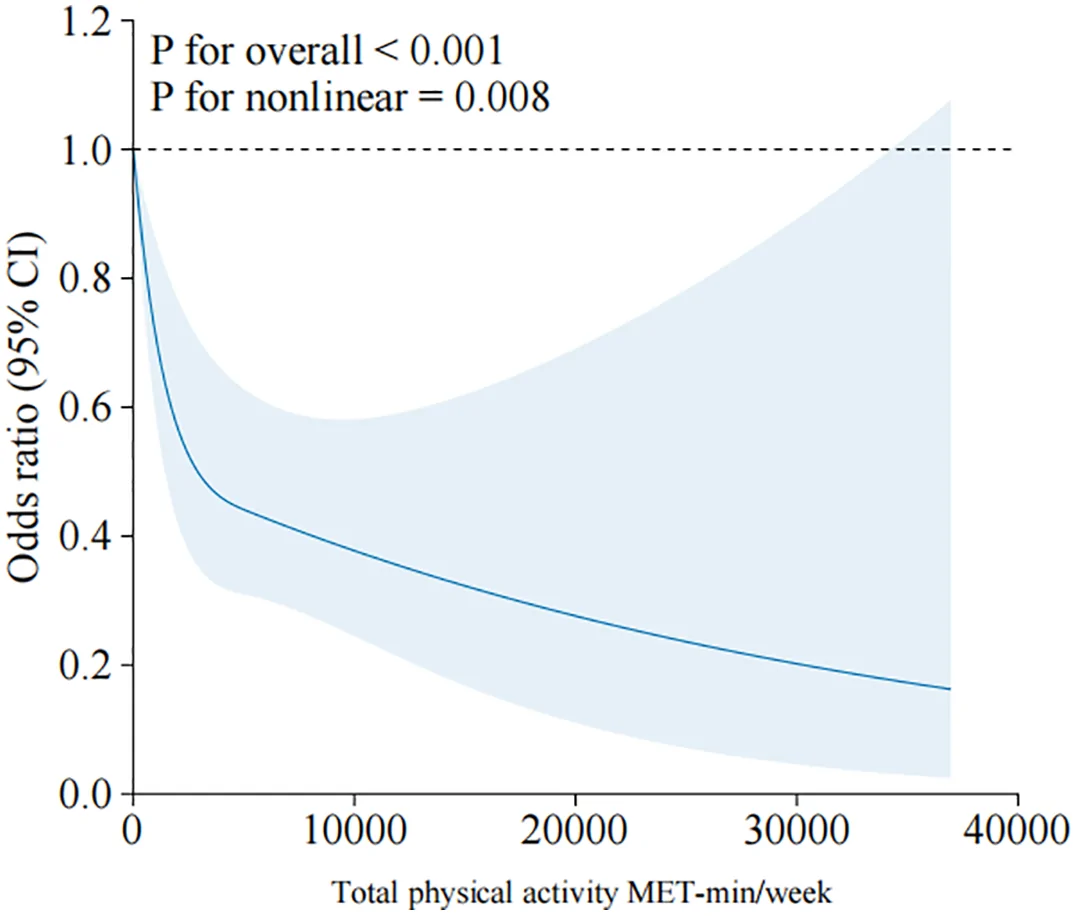
Restricted cubic spline analysis of the association between total physical activity and self-reported emphysema. Total physical activity was modeled as a continuous variable using a restricted cubic spline with three knots. The solid blue line represents the estimated odds ratio, and the shaded area indicates the corresponding 95% confidence interval. The horizontal dashed line denotes an odds ratio of 1. Total physical activity is expressed in metabolic equivalent of task minutes per week (MET-min/week). The P values for the overall association and nonlinearity were <0.001 and 0.008, respectively.

### Subgroup analysis

The subgroup analysis of the association between PA levels and self-reported emphysema, stratified by sex, alcohol consumption, smoking status, and BMI, is shown in Fig 3. Among males, participants engaging in the highest PA category (≥3000 MET-min/week) exhibited a significantly lower odds of self-reported emphysema (OR, 0.30; 95% CI, 0.15–0.60; p = 0.001), whereas no significant association was observed among females (p for interaction = 0.17). Similarly, alcohol consumers in the highest PA category showed an inverse association (OR, 0.34; 95% CI, 0.19–0.61; p = 0.001), while no significant association was identified in non-drinkers (p for interaction = 0.118).

**Fig 3:**
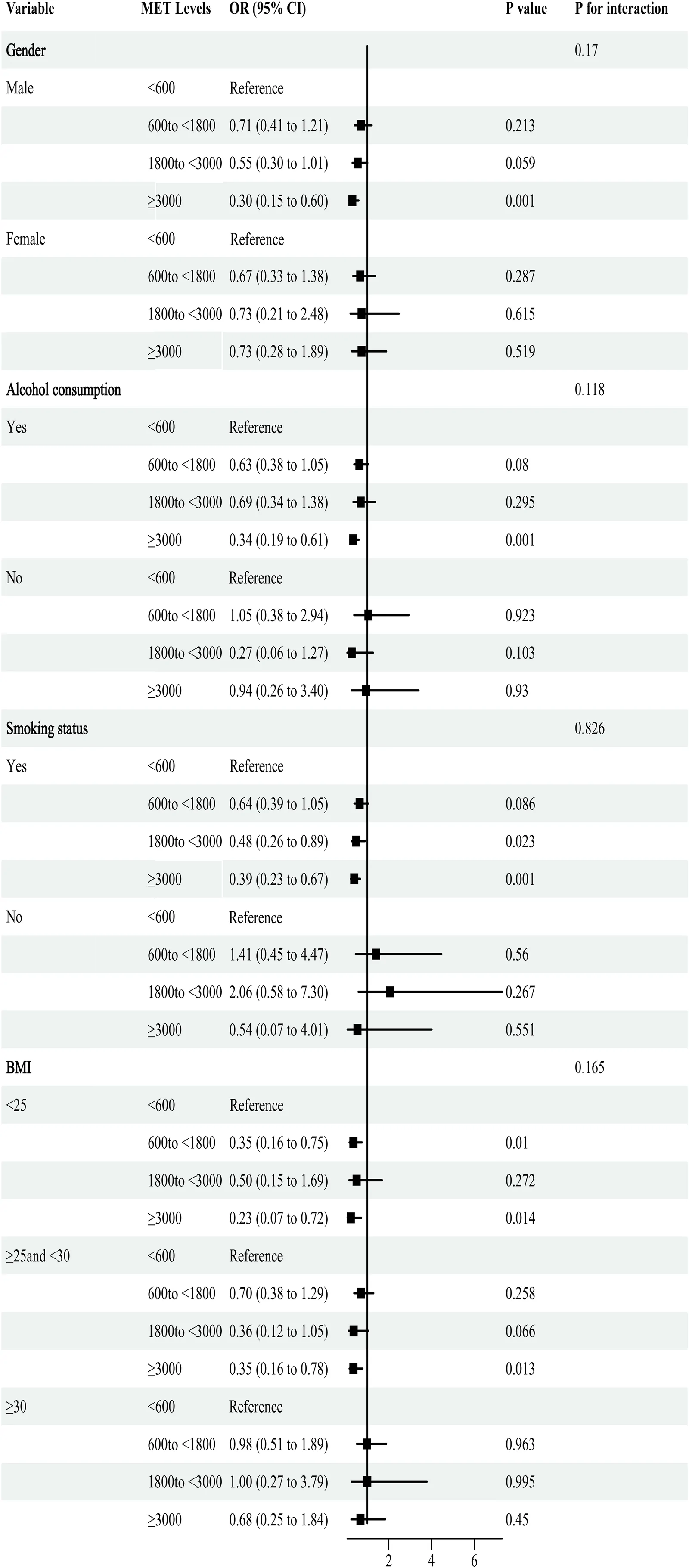
Subgroup analyses of the association between physical activity and self-reported emphysema. Associations are presented by sex, alcohol consumption, smoking status, and body mass index (BMI). Within each subgroup, total physical activity was categorized as <600, 600 to <1,800, 1,800 to <3,000, and ≥3,000 metabolic equivalent of task minutes per week (MET-min/week), with <600 MET-min/week as the reference category. Squares represent odds ratios (ORs), and horizontal lines indicate 95% confidence intervals (CIs). The vertical reference line denotes an OR of 1. P values for interaction assess whether the association between physical activity and self-reported emphysema differs across strata of each subgroup variable. BMI categories are expressed in kg/m².

The inverse association between higher PA levels and self-reported emphysema was more pronounced among smokers, with an OR of 0.39 (95% CI, 0.23–0.67; p = 0.001) for participants achieving ≥3000 MET-min/week. In contrast, no significant association was observed among non-smokers (p for interaction = 0.826). BMI-specific analyses revealed that participants with a BMI < 25 had lower odds of self-reported emphysema at higher PA levels, particularly those in the ≥ 3000 MET-min/week category (OR, 0.23; 95% CI, 0.07–0.72; p = 0.014). However, this association was attenuated among overweight and obese individuals (p for interaction = 0.165).

These findings suggest heterogeneity in the association between PA and self-reported emphysema, with the stronger inverse associations observed among males, alcohol consumers, smokers, and individuals with a lower BMI.

To assess the robustness and generalizability of the primary findings, an external validation analysis was performed using data from the China Health and Retirement Longitudinal Study (CHARLS 2015, n = 4,496). As summarized in Table 3, participants with higher levels of physical activity (PA) were generally younger, more likely to be married, and exhibited lower prevalence of hypertension and self-reported lung disease (both p < 0.05).

**Table 3:** Baseline characteristics of participants by total physical activity (CHARLS 2015).

Characteristic	Total physical activity MET-min/week					
	Total (N = 4496)	<600 MET (n = 941)	600– < 1800 (n = 795)	1800– < 3000 (n = 286)	≥3000 (n = 2474)	p Value
**Age, year (mean ± SD)**	68.6 ± 7.0	70.5 ± 7.7	69.4 ± 7.0	67.6 ± 6.0	66.9 ± 5.8	<0.001
**Sex (male, n (%))**	2,241 (49.86%)	421 (44.73%)	396 (49.81%)	132 (46.15%)	1,292 (52.21%)	0.001
**Education level (≥high school, n (%))**	4,133 (91.92%)	901 (95.74%)	696 (87.55%)	245 (85.66%)	2,291 (92.60%)	<0.001
**Married/living with partner (n (%))**	3,659 (81.41%)	683 (72.57%)	624 (78.49%)	239 (83.57%)	2,113 (85.40%)	<0.001
**High income (upper PIR tertile, n (%))**	1,747 (38.86%)	222 (23.60%)	127 (15.97%)	13 (4.55%)	1,385 (56.00%)	<0.001
**BMI, kg/m² (mean ± SD)**	23.5 ± 4.0	23.5 ± 4.2	23.7 ± 4.1	23.7 ± 3.8	23.4 ± 4.0	0.148
**BMI ≥ 30 (n (%))**	1,599 (35.59%)	238 (25.29%)	162 (20.38%)	19 (6.64%)	1,180 (47.69%)	<0.001
**Alcohol use (yes, n (%))**	1,487 (33.07%)	224 (23.81%)	218 (27.42%)	95 (33.22%)	950 (38.41%)	<0.001
**Smoking (yes, n (%))**	2,123 (47.26%)	417 (44.31%)	367 (46.16%)	136 (47.55%)	1,203 (48.65%)	0.233
**Total cholesterol (mg/dL, mean ± SD)**	187.6 ± 37.8	186.9 ± 37.8	186.9 ± 38.7	189.5 ± 34.4	185.1 ± 35.3	0.314
**Triglycerides (mg/dL, mean ± SD)**	142.9 ± 88.2	141.9 ± 89.2	143.8 ± 85.8	152.6 ± 88.7	137.2 ± 87.3	0.061
**Creatinine (mg/dL, mean ± SD)**	0.84 ± 0.34	0.82 ± 0.29	0.87 ± 0.48	0.84 ± 0.25	0.82 ± 0.24	0.019
**Hypertension (yes, n (%))**	1,727 (38.41%)	441 (46.89%)	328 (41.26%)	109 (38.11%)	849 (34.32%)	<0.001
**Diabetes (yes, n (%))**	678 (15.08%)	219 (23.28%)	154 (19.37%)	23 (8.04%)	282 (11.40%)	0.063
**Self-reported lung disease** **(yes, n (%))**	520 (11.57%)	231 (24.56%)	146 (18.36%)	19 (6.64%)	124 (5.01%)	0.027

In multivariable logistic regression analyses (Table 4), higher PA levels were inversely associated with lung disease. Participants achieving ≥3000 MET-min/week demonstrated significantly lower odds of lung disease (OR = 0.64; 95% CI: 0.46–0.89; p = 0.008). The dose–response relationship across PA categories remained significant (p for trend = 0.008), consistent in direction and magnitude with the NHANES findings.

**Table 4:** Logistic regression of Physical Activity and Lung Disease (CHARLS 2015).

Physical activity (MET-min/week)	Model 1		Model 2		Model 3	
	OR (95% CI)	P-Value	OR (95% CI)	P-Value	OR (95% CI)	P-Value
<600	Reference		Reference		Reference	
600– < 1800	0.89 (0.69–1.14)	0.345	0.89 (0.69–1.14)	0.346	0.87 (0.58–1.29)	0.481
1800– < 3000	0.86 (0.60–1.21)	0.381	0.91 (0.64–1.30)	0.599	0.74 (0.41–1.33)	0.313
≥3000	0.75 (0.61–0.91)	0.003	0.79 (0.64–0.96)	0.021	0.64 (0.46–0.89)	0.008
P for trend	0.028		<0.001		0.008	

Restricted cubic spline and subgroup analyses were not repeated in the validation cohort due to the limited number of self-reported lung disease cases. Nevertheless, the consistent direction and comparable effect size across both populations support the external validity and robustness of the observed associations.

## Discussion

Our analysis of the NHANES database shows that higher intensity physical activity (exceeding 3000 MET-minutes per week) is associated with lower odds of self-reported emphysema, with a clear dose-response relationship. This finding suggests that physical activity may be an important correlate of self-reported emphysema and support its potential relevance in public health strategies related to pulmonary diseases.

Our findings are consistent with previous research, which has indicated that regular physical activity can improve both physiological and psychological health conditions in patients with Chronic Obstructive Pulmonary Disease (COPD). For instance, Albarrati et al. found that moderate to high-intensity physical activity was significantly associated with lower odds of COPD exacerbations [16–18]. In addition, accumulating evidence suggests that physical activity is inversely associated with sedentary behavior, which in turn has been linked to adverse health outcomes, including several types of cancers [19]. Our study further quantifies the association between physical activity and self-reported emphysema, particularly at higher activity levels, adding to the existing body of observational evidence.

To enhance the robustness of our findings, we conducted an external validation using data from the China Health and Retirement Longitudinal Study (CHARLS). Despite differences in population structure, lifestyle, and ethnicity between the U.S. (NHANES) and Chinese (CHARLS) cohorts, the inverse association between higher physical activity and self-reported emphysema in NHANES and self-reported lung disease in CHARLS was consistent, with comparable effect sizes and a significant dose-response trend. This external validation supports the generalizability of our results across distinct populations.

Several biological mechanisms may help explain the observed associations. Regular physical activity has been associated with improved respiratory muscle function, enhanced cardiopulmonary efficiency, and reduced systemic inflammation [20–23]. In addition, physical activity may contribute to improved oxidative stress balance, mitochondrial function, and metabolic regulation [24–26]. These mechanisms may help explain the observed associations between physical activity and emphysema. Studies have shown that aerobic exercise is associated with improvements in emphysema and pulmonary fibrosis in mice with COPD by reducing inflammatory responses and oxidative stress damage [27]. Finally, physical activity has been associated with improved metabolic health, including lower body weight and enhanced insulin sensitivity [28], which may be relevant for COPD patients who often have comorbidities such as cardiovascular diseases. The combined effects of these mechanisms not only provide a scientific basis for the clinical application of physical activity but also guide future research directions.

Through these mechanisms, physical activity may be associated with better physiological status and improved quality of life among individuals with emphysema. From a biological standpoint, physical activity has been suggested to be linked to preservation of lung parenchymal integrity [29,30]. Enhanced respiratory muscle performance and improved thoracic compliance may be associated with reduced dynamic hyperinflation and maintenance of alveolar architecture [31]. Moreover, physical activity has been associated with lower levels of systemic and pulmonary oxidative stress, decrease levels of pro-inflammatory cytokines, and inhibit apoptosis in lung tissues—processes that contribute to the structural changes in emphysema [32]. These observations support the plausibility of the associations identified in this study and suggest that physical activity may represent a relevant non-pharmacological factor in lung health.

This study has several limitations. The cross-sectional design limits our ability to infer causality, and future research should adopt a prospective cohort design to better evaluate the association between physical activity and self-reported emphysema. Moreover, reverse causation cannot be excluded, as individuals with undiagnosed or early-stage emphysema may have already reduced their physical activity levels due to subclinical respiratory symptoms, thereby influencing the observed associations. Additionally, although we adjusted for a wide range of sociodemographic, behavioral, and clinical covariates, the possibility of residual confounding remains. Factors such as environmental exposures (e.g., air pollution), occupational hazards, or genetic susceptibility were not captured in the dataset and may have influenced the results. Since the physical activity data relies on self-reporting, there is a potential for recall or reporting bias. The use of objective measurement tools would help improve the accuracy of the data and the reliability of the research. Furthermore, although we employed a robust modeling strategy—including stepwise covariate adjustment, restricted cubic spline analysis, and subgroup stratification—we did not incorporate formal model diagnostics such as Akaike Information Criterion (AIC), Bayesian Information Criterion (BIC), or multicollinearity indices. Given the large sample size and biologically grounded covariate selection, we believe the potential impact of omitted diagnostics is limited. Nevertheless, this remains a methodological limitation that should be considered when interpreting our findings.

In addition, while the CHARLS cohort used for external validation relied on self-reported lung disease and had different demographic characteristics from the NHANES population, the consistent direction and magnitude of associations observed between physical activity and lung disease are consistent with the robustness and generalizability of our findings. Future multicenter longitudinal studies using objective activity monitoring and standardized diagnostic criteria are warranted to further evaluate these associations and clarify potential causal pathways.

These findings may have implications for public health strategies targeting populations at higher risk, particularly in informing policy development and population-level health promotion efforts. Public health authorities may consider promoting appropriate levels of physical activity as part of broader health initiatives. Future research should explore the associations of different types and intensities of physical activity with specific COPD subtypes. Moreover, factors such as gender, age, ethnicity, and socioeconomic status may influence these associations and should be further investigated in future studies.

## Conclusion

This study systematically assessed the association between physical activity levels and self-reported emphysema using data from the NHANES database. The results indicate that higher levels of physical activity (exceeding 3000 MET-minutes per week) were associated with lower odds of self-reported emphysema, with a clear dose–response pattern. These findings suggest a potential role of physical activity in lung health at the population level.

Although the cross-sectional design of this study limits causal inference, the observed association between higher physical activity levels and lower odds of self-reported emphysema provides a basis for further prospective research. Future studies using objective activity monitoring and longitudinal follow-up are needed to further evaluate these associations and explore underlying biological mechanisms.

## References

[1] Gao Y-H, Guan W-J, Liu Q, Wang H-Q, Zhu Y-N, Chen R-C, et al. Impact of COPD and emphysema on survival of patients with lung cancer: A meta-analysis of observational studies. Respirology. 2016;21(2):269–79. doi: 10.1111/resp.12661 26567533

[2] Ferrera MC, Labaki WW, Han MK. Advances in Chronic Obstructive Pulmonary Disease. Annu Rev Med. 2021;72:119–34. doi: 10.1146/annurev-med-080919-112707 33502902 PMC8011854

[3] Callejón-Leblic B, Sánchez Espirilla S, Gotera-Rivera C, Santana R, Díaz-Olivares I, Marín JM. Metallomic Signatures of Lung Cancer and Chronic Obstructive Pulmonary Disease. Int J Mol Sci. 2023;24:14250. doi: 10.3390/ijms241814250 37762552 PMC10532173

[4] Mouronte-Roibás C, Leiro-Fernández V, Fernández-Villar A, Botana-Rial M, Ramos-Hernández C, Ruano-Ravina A. COPD, emphysema and the onset of lung cancer. A systematic review. Cancer Lett. 2016;382(2):240–4. doi: 10.1016/j.canlet.2016.09.002 27666776

[5] Shah PL, Herth FJ, van Geffen WH, Deslee G, Slebos D-J. Lung volume reduction for emphysema. Lancet Respir Med. 2017;5(2):147–56. doi: 10.1016/S2213-2600(16)30221-1 27693408

[6] Ries AL, Make BJ, Reilly JJ. Pulmonary rehabilitation in emphysema. Proc Am Thorac Soc. 2008;5(4):524–9. doi: 10.1513/pats.200707-093ET 18453366 PMC2645330

[7] Li Z, Liu S, Wang L, Smith L. Mind-Body Exercise for Anxiety and Depression in COPD Patients: A Systematic Review and Meta-Analysis. Int J Environ Res Public Health. 2019;17(1):22. doi: 10.3390/ijerph17010022 31861418 PMC6981896

[8] Janczura M, Bochenek G, Nowobilski R, Dropinski J, Kotula-Horowitz K, Laskowicz B. The Relationship of Metabolic Syndrome with Stress, Coronary Heart Disease and Pulmonary Function--An Occupational Cohort-Based Study. PLoS One. 2015;10:e0133750. doi: 10.1371/journal.pone.0133750 26274823 PMC4537246

[9] Vilar-Gomez E, Nephew LD, Vuppalanchi R, Gawrieh S, Mladenovic A, Pike F, et al. High-quality diet, physical activity, and college education are associated with low risk of NAFLD among the US population. Hepatology. 2022;75(6):1491–506. doi: 10.1002/hep.32207 34668597

[10] Afzal M, Greco F, Quinzi F, Scionti F, Maurotti S, Montalcini T, et al. The Effect of Physical Activity/Exercise on miRNA Expression and Function in Non-Communicable Diseases-A Systematic Review. Int J Mol Sci. 2024;25:6813. doi: 10.3390/ijms25136813 38999923 PMC11240922

[11] Jessup M, Reza N. Walking Away From Heart Failure. JACC Heart Fail. 2018;6(12):996–8. doi: 10.1016/j.jchf.2018.07.005 30196072

[12] Pate RR, Pratt M, Blair SN, Haskell WL, Macera CA, Bouchard C, et al. Physical activity and public health. A recommendation from the Centers for Disease Control and Prevention and the American College of Sports Medicine. JAMA. 1995;273(5):402–7. doi: 10.1001/jama.273.5.402 7823386

[13] Liu H, Wang L, Chen C, Dong Z, Yu S. Association between dietary niacin intake and migraine among american adults: National Health and Nutrition Examination Survey. Nutrients. 2022;14(15):3052. doi: 10.3390/nu14153052 35893904 PMC9330821

[14] Fryar CD, Ostchega Y, Hales CM, Zhang G, Kruszon-Moran D. Hypertension prevalence and control among adults: United States, 2015-2016. 2017. 29155682

[15] Menke A, Casagrande S, Geiss L, Cowie CC. Prevalence of and trends in diabetes among adults in the United States, 1988-2012. JAMA. 2015;314:1021–9. doi: 10.1001/jama.2015.10029 26348752

[16] Albarrati AM, Gale NS, Munnery MM, Cockcroft JR, Shale DJ. Daily physical activity and related risk factors in COPD. BMC Pulm Med. 2020;20(1):60. doi: 10.1186/s12890-020-1097-y 32138714 PMC7059270

[17] Sievi NA, Kohler M, Thurnheer R, Leuppi JD, Irani S, Frey M, et al. No impact of exacerbation frequency and severity on the physical activity decline in COPD: a long-term observation. Int J Chron Obstruct Pulmon Dis. 2019;14:431–7. doi: 10.2147/COPD.S188710 30863043 PMC6388775

[18] Ramon MA, Esquinas C, Barrecheguren M, Pleguezuelos E, Molina J, Quintano JA, et al. Self-reported daily walking time in COPD: relationship with relevant clinical and functional characteristics. Int J Chron Obstruct Pulmon Dis. 2017;12:1173–81. doi: 10.2147/COPD.S128234 28458527 PMC5402919

[19] Kerr J, Anderson C, Lippman SM. Physical activity, sedentary behaviour, diet, and cancer: an update and emerging new evidence. Lancet Oncol. 2017;18(8):e457–71. doi: 10.1016/S1470-2045(17)30411-4 28759385 PMC10441558

[20] Tomas-Carus P, Biehl-Printes C, Del Pozo-Cruz J, Parraca JA, Folgado H, Pérez-Sousa MÁ. Effects of respiratory muscle training on respiratory efficiency and health-related quality of life in sedentary women with fibromyalgia: a randomised controlled trial. Clin Exp Rheumatol. 2022;40(6):1119–26. doi: 10.55563/clinexprheumatol/0v55nh 35748715

[21] Sperlich B, Fricke H, de Marées M, Linville JW, Mester J. Does respiratory muscle training increase physical performance?. Mil Med. 2009;174(9):977–82. doi: 10.7205/milmed-d-04-6408 19780375

[22] Paez PN, Phillipson EA, Masangkay M, Sproule BJ. The physiologic basis of training patients with emphysema. Am Rev Respir Dis. 1967;95(6):944–53. doi: 10.1164/arrd.1967.95.6.944 6067380

[23] Watz H, Waschki B, Boehme C, Claussen M, Meyer T, Magnussen H. Extrapulmonary effects of chronic obstructive pulmonary disease on physical activity: a cross-sectional study. Am J Respir Crit Care Med. 2008;177(7):743–51. doi: 10.1164/rccm.200707-1011OC 18048807

[24] Zhou Y, Jia N, Ding M, Yuan K. Effects of exercise on inflammatory factors and IGF system in breast cancer survivors: a meta-analysis. BMC Womens Health. 2022;22(1):507. doi: 10.1186/s12905-022-02058-5 36482346 PMC9730577

[25] Liu L, Tang J, Liang X, Li Y, Zhu P, Zhou M, et al. Running exercise alleviates hippocampal neuroinflammation and shifts the balance of microglial M1/M2 polarization through adiponectin/AdipoR1 pathway activation in mice exposed to chronic unpredictable stress. Mol Psychiatry. 2024;29(7):2031–42. doi: 10.1038/s41380-024-02464-1 38361125

[26] Angulo J, El Assar M, Álvarez-Bustos A, Rodríguez-Mañas L. Physical activity and exercise: Strategies to manage frailty. Redox Biol. 2020;35:101513. doi: 10.1016/j.redox.2020.101513 32234291 PMC7284931

[27] Wang X, Wang Z, Tang D. Aerobic Exercise Alleviates Inflammation, Oxidative Stress, and Apoptosis in Mice with Chronic Obstructive Pulmonary Disease. Int J Chron Obstruct Pulmon Dis. 2021;16:1369–79. doi: 10.2147/COPD.S309041 34040365 PMC8139730

[28] Friedenreich CM, Ryder-Burbidge C, McNeil J. Physical activity, obesity and sedentary behavior in cancer etiology: epidemiologic evidence and biologic mechanisms. Mol Oncol. 2021;15(3):790–800. doi: 10.1002/1878-0261.12772 32741068 PMC7931121

[29] Brandao-Rangel M, Brill B, de Souza Carvalho E, Melamed D, Moraes-Ferreira R, Silva-Reis A, et al. Physically Active Lifestyle Attenuates Impairments on Lung Function and Mechanics in Hypertensive Older Adults. Adv Respir Med. 2024;92:278–90. doi: 10.3390/arm92040027 39051189 PMC11270291

[30] Puente-Maestu L, Stringer WW. Physical activity to improve health: do not forget that the lungs benefit too. Eur Respir J. 2018;51(2):1702468. doi: 10.1183/13993003.02468-2017 29386339

[31] Petrovic M, Reiter M, Zipko H, Pohl W, Wanke T. Effects of inspiratory muscle training on dynamic hyperinflation in patients with COPD. Int J Chron Obstruct Pulmon Dis. 2012;7:797–805. doi: 10.2147/COPD.S23784 23233798 PMC3516469

[32] Toledo AC, Magalhaes RM, Hizume DC, Vieira RP, Biselli PJC, Moriya HT, et al. Aerobic exercise attenuates pulmonary injury induced by exposure to cigarette smoke. Eur Respir J. 2012;39(2):254–64. doi: 10.1183/09031936.00003411 21700603

